# The Preliminary Efficacy of a Program to Facilitate Benefit Finding for People with Mental Illness: A Pilot Randomized Controlled Trial in Japan

**DOI:** 10.3390/healthcare10081491

**Published:** 2022-08-08

**Authors:** Rie Chiba, Akiko Funakoshi, Yuki Miyamoto

**Affiliations:** 1 Department of Nursing, Graduate School of Health Sciences, Kobe University, Kobe 654-0142, Japan; 2 Division of Psychiatric and Mental Health Nursing, Department of Nursing, Kobe City College of Nursing, Kobe 651-2103, Japan; 3 Department of Psychiatric Nursing, Graduate School of Medicine, The University of Tokyo, Tokyo 113-0033, Japan

**Keywords:** benefit finding, feasibility, intervention, Japan, mental illness, personal recovery, pilot randomized controlled trial

## Abstract

Benefit finding is a concept that refers to finding positive changes or benefits through negative experiences from stressful life events. The present study aimed to develop a new intervention program to facilitate benefit finding for people with mental illness and examine its feasibility and preliminary efficacy from pilot data. We hypothesized that participants who joined the group-based intervention program would show progress in benefit finding, personal recovery, and well-being, as well as alleviated psychiatric symptoms and functional impairment, compared to participants in the control group. The participants in the intervention group joined in a new program which focuses on (1) cognitive–behavioral stress management and (2) own experiences, including what was found or realized through their lives since the onset of mental illness. The program used a workbook comprised of eight 90-min sessions, with one held every week. Twenty-four were found eligible and provided informed consent to participate in the study. About 46% were males, and the average age was 42.5 years. Around 63% were diagnosed with schizophrenia. We did not find significant differences over time by groups. However, medium to large effects in each scale or at least one subscale (i.e., benefit finding, personal recovery, subjective well-being, and psychiatric symptoms and functional impairment) were observed. Future studies with more participants from various settings would be necessary to exactingly examine the effectiveness of the intervention program.

## 1. Introduction

Mental illness often has chronic and persistent courses with repeated remission and deterioration, which causes critical impacts on the quality of life. Therefore, not only aiming for cure or illness alleviation is important but also supporting a person to rebuild a fulfilling and satisfying life even if the symptoms are not perfectly cured [1,2,3]. Benefit finding is a concept that refers to finding positive changes or benefits through negative experiences from stressful life events, such as chronic illness [4]. Some researchers interpret it as a positively oriented emotional coping strategy in adversity. Hence, benefit finding is described as a positive reappraisal process, which can foster positive emotions and behaviors among people who are going through life-changing experiences [5,6]. Particularly, it has also been conceptualized as a process of positive meaning making [7]. An earlier qualitative study in the psychiatric field on benefit finding among Japanese people with mental illness revealed several themes related to strengthened relationships with others, personal change of life values, health-related behavioral changes, increased understanding of mental illness, and finding a new role in society [8]. As another concept, personal recovery in psychiatric rehabilitation is a concept described as a complex process of developing new meaning and life purpose as people grow beyond the catastrophic effects of mental illness [9]. Rather than the traditional medical model that emphasizes returning to a previous mental health state, it has become a core concept in mental health services, treatment, and policies [1,2].

While benefit finding and personal recovery have similar perspectives, benefit finding is explained as a phenomenon relevant to coping, which can be enhanced in people with chronic illness, such as cancer [10,11]. On the other hand, personal recovery indicates a subjective process of their own lives with mental illness. Previous studies suggested that the experience of benefit finding would contribute to further progress in personal recovery among people with mental illness. Particularly, enhancing benefit finding may lead to higher personal recovery as well as subjective well-being and better mental health [7,12,13,14,15]. Therefore, many studies to date have emphasized the significance to facilitate benefit finding, including for people with mental illness [8,13,14]. However, few intervention studies have been conducted on people with mental illness, while benefit finding among people with mental illness has been drawing attention in recent years [13,14].

Earlier studies suggested several methodologies to enhance benefit finding. Some intervention studies for cancer patients suggested a cognitive–behavioral stress management approach as effective to enhance benefit finding [16,17,18]. Another principle underpinned is to think and express their experiences of benefit finding [13,19,20]. To our knowledge, studies have not been published which examined the effects of an intervention that combined these two methodological frameworks, although the number of intervention studies to facilitate benefit finding has been increasing recently.

Therefore, this study aimed to develop a new intervention program to facilitate benefit finding for people with mental illness and examine its feasibility and preliminary efficacy from pilot data on key outcomes (benefit finding, personal recovery, subjective well-being, and psychiatric symptoms and functional impairment). It is the first intervention study to newly develop an intervention program including two different approaches to facilitate benefit finding. This study was placed as a preliminary research for future large-scale multi-site RCT. We hypothesized that participants who joined the intervention program would show progress in benefit finding, personal recovery, and well-being, as well as alleviated psychiatric symptoms and functional impairment, compared to other participants who did not join the program.

## 2. Materials and Methods

### 2.1. Program Development to Facilitate Benefit Finding: “To Live Lively”

This study developed a new program to facilitate benefit finding, which focuses on (1) cognitive–behavioral stress management and (2) own experiences, including what was found or realized through their lives since the onset of mental illness.

First, the cognitive–behavioral stress management section was developed with reference to earlier studies that aimed to increase benefit finding [16,17,18]. The stress management section included components, such as stressors and awareness, cognitive distortion and rational thought, coping, social support, and breathing.

Second, the own experience section was developed with reference to the framework for facilitating post-traumatic growth [21,22]. This section included listening to the personal experience and benefit finding of a peer with mental illness, reviewing one’s struggle with adversity that results from mental illness, knowing about the types of benefit finding that could be experienced by individuals with mental illness, and thinking about one’s benefit finding. The first author made a workbook for this study for the participants to know the information regarding the contents and write their thoughts, feelings, and experiences. Another two authors and two peers with mental illness checked the workbook for parts that are difficult to understand or need to be revised.

Overall, the group-based program used a workbook comprised of eight 90-min sessions with one held every week. The introduction was held as 0.5 sessions, the stress management section was held as 3.5 sessions, followed by the next section as 3.0 sessions, with a wrap up as 1.0 session. Additionally, breathing and daily mental health condition self-check were included as homework. The program was designed to be held in a group of 7–8 participants facilitated by a healthcare professional and a peer worker with mental illness. To ensure the principle and content of the program, as well as the fidelity, the first author made a facilitation guide and a checklist for the facilitators to confirm whether each session was conducted properly.

### 2.2. Preliminary Pre–Post Study

Before the current randomized control trial, a preliminary pre–post study was conducted to check any improvements in the workbook or the interventional program implementation as well as whether there were any side effects such as psychological burden. A preliminary pre–post study was held on seven individuals with mental illness at a social welfare facility for psychiatric rehabilitation from March to April 2018. The first author (R.C.) has enough clinical experiences as a psychiatric nurse and an expertise to conduct this intervention. Before the pre–post study, the first author and a peer supporter with many experiences of peer support had meetings to ensure how to proceed each session based on the facilitation guide. In addition, we had meetings before and after each session to check how we proceed the session and whether we secured the fidelity. Any difficulties in implementation and how they were dealt with were discussed with each other. The first author and a peer supporter with mental illness facilitated eight sessions, and the first author asked for feedback from the participants after each session. Some words and expressions in the workbook were revised based on the feedback from the participants. The program was considered to not cause any inconvenience or side effects to the participants. A single-arm pre–post test also revealed the potential to enhance benefit finding. Therefore, we decided to proceed to the subsequent pilot randomized controlled trial to examine in detail the feasibility and effectiveness.

### 2.3. Participants and Randomization

Participants were recruited from four social rehabilitation facilities for welfare employment in the Kansai region in Japan and were held from August to September 2018 at one facility and in January 2019 at the other three facilities.

Subjects for whom permission to contact was obtained from facility staff were assessed for eligibility according to the following criteria: (1) diagnosed by a psychiatrist with mental illness, (2) 20 years old or above, (3) community residence, and (4) service user at one of the four facilities. The required sample size to detect an expected effect size of 0.25 (medium to large), with a power of 0.8 and α of 0.05 (two-tailed), was calculated as 38 with an assumed 20% dropout rate, by G*Power 3 [23]. 

Using a random number table that was generated by a co-researcher, the randomized allocation was completed. A co-researcher independently generated and accordingly assigned these to the participants. The researchers knew to which of the groups the individuals belonged.

### 2.4. Procedures

The intervention group underwent the weekly program named “To live lively” in eight 90-min group sessions over 2 months. One intervention group from one facility was held from September to November 2018, whereas another intervention group was held from February to March 2019. Each session was collaboratively facilitated by a psychiatric nurse and a peer co-facilitator with mental illness. After each session, those in the intervention group were asked whether they felt any side effects such as psychological burden. Fidelity was monitored through a checklist by the first author and the peer supporter at the end of each session.

The control group was provided with services as usual at each facility during the same period. A self-administered questionnaire survey for all participants was conducted three times, i.e., at baseline (T1), after intervention (8 weeks from baseline) (T2), and 3 months after intervention (approximately 20 weeks from baseline) (T3).

### 2.5. Measures

#### 2.5.1. Benefit Finding Questionnaire (BFQ)

The BFQ is a scale that was developed in Japan to assess one’s degree of experience in benefit finding [24]. It comprises two domains, including changes in the sense of values and way of thinking, and changes in relationships with others. Items, such as “Your ties (relationships) with your family have been…” are rated on a 5-point Likert scale from 1 to 5, with higher total scores indicating further experiences in benefit finding. Good reliability and validity were confirmed [24].

#### 2.5.2. Recovery Assessment Scale (RAS)

The RAS assesses personal recovery among people with mental illness [25]. It includes 24 items, such as “I have goals in life that I want to reach”. Items are rated on a 5-point Likert scale ranging from 1 to 5, with higher total scores indicating further progress in recovery. Factor analyses in a Japanese study showed five factors, including goal/success orientation and hope, reliance on others, personal confidence, no domination by symptoms, and willingness to ask for help. Its good reliability and validity have been confirmed [26].

#### 2.5.3. World Health Organization (WHO)-5 Well-Being Index

The WHO-5 is one of the most sensitive and valid scales of subjective well-being [27]. The WHO-5 includes five items, such as “I have felt cheerful and in good spirits”. The respondent is asked to rate how well each statement applies to themselves, considering the last 14 days. Each item is scored from 0 (none of the time) to 5 (all of the time). The Japanese version of WHO-5 (WHO-5-J) showed its good reliability and validity [28].

#### 2.5.4. Behavior and Symptom Identification Scale (BASIS-32)

BASIS-32 is a 32-item scale that assesses psychiatric symptoms and functional impairment in the past week [29]. It has five domains that represent the relationship to self and others; daily living and role functioning; depression and anxiety; impulsive and addictive behavior; and psychosis. Each item, such as “Managing day-to-day life (e.g., getting to places on time, handling money, making everyday decisions)”, is rated on a 5-point Likert scale ranging from 0 (no difficulty) to 4 (extreme difficulty), with higher total scores indicating more severe psychiatric symptoms and functional impairment. The reliability and validity of the Japanese version of BASIS-32 have been confirmed [30].

### 2.6. Statistical Analysis

The mean and standard deviation were calculated for each demographic and clinical variable at baseline, and outcome variables were calculated as well at three time points (baseline, post-intervention, and follow-up) in the intervention and control groups. The box plots were also displayed. Due to a quite small sample size, the *t*-test, instead of repeated ANOVA, was used to examine the differences in the scores times (T2 and T1, as well as T3 and T1) between groups. Effect sizes were also calculated. Effect sizes, *d* = 0.15, 0.40, and 0.75, were interpreted as small, medium, or large, respectively [31]. An intention-to-treat (ITT) analysis was performed. All participants were included regardless of the number of participations, whether they dropped out or completed the questionnaire. Statistical analysis was performed using the IBM Statistical Package for the Social Sciences Statistics (SPSS) version 25 for Windows (IBM Inc., New York, NY, USA). *p*-values of <0.05 were considered statistically significant (two-tailed).

### 2.7. Ethical Considerations and Clinical Trial Register

To secure appropriate ethical standards, the candidates were informed both in writing and orally regarding the purpose and methods of the study as well as the data storage and privacy protection methods. They were also informed that non-participants would not be disadvantaged, and participants were allowed to withdraw from the study any time. Participants who consented to participate in the study provided a researcher with written informed consent before the randomization. Data obtained in this study were handled and analyzed with appropriate precautions.

The preliminary pre–post study was approved by the ethical committee of University of Hyogo (No. KYOIN23) in 2018. A subsequent pilot randomized controlled trial was approved by the ethical committee of University of Hyogo (No. KYOIN5) in 2018 and Kobe University (No. 826) in 2019. 

The CONSORT Statement [32] was referred to for the study protocol of this randomized controlled trial. The research protocol was registered on UMIN-CTR (No. R000038630) before the study onset.

## 3. Results

**Figure 1:**
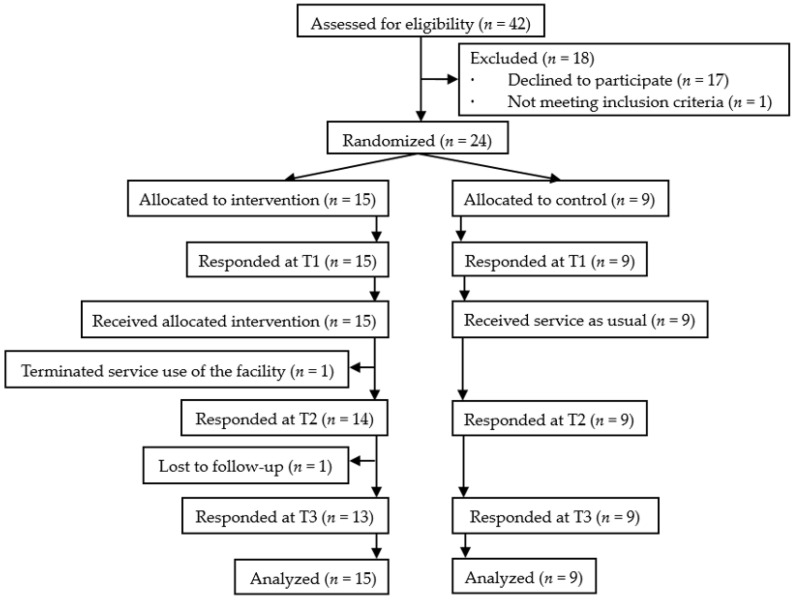
**CONSORT flow diagram** of the participants. T1: Baseline; T2: Post-intervention (eight weeks after baseline); T3: Three-month follow-up after the post-intervention.

Among a total of 42 subjects contacted, 24 were found eligible and provided informed consent to participate in the study. Some candidates did not agree to participate in the study due to the inconvenient schedule, unstable mental state, or their plans to start to work in new companies soon. Therefore, 24 persons were randomly allocated to the intervention group (*n* = 15) or the control group (*n* = 9) (Figure 1).

Table 1 shows the sociodemographic and clinical characteristics of the participants. Of the participants, 45.8% were males. The mean age was 42.5 years old (standard deviation = 12.9), with a range between 20 and 76. The average duration of mental illness was 16.3 years (standard deviation = 9.9). Of the participants, 62.5% were diagnosed with schizophrenia, which was followed by depression in 25.0% and bipolar disorder in 8.3%. One-quarter had experienced hospitalization in psychiatric wards. Three-quarters of the participants used employment support services, and one-third of the participants were involved in activities as a peer to help other people with mental illness. Figure 1 shows that 2/15 participants (seven allocated into the intervention group) dropped out from the study, of whom one terminated service at the facility before the post-intervention survey, and another one rarely came to the facility after the post-intervention survey. The remaining 10 participants completed the study. No one in the intervention group answered that they experienced a psychological burden through the sessions.

Statistically significant differences in score changes were not found (T2 and T1, T3 and T1) between the groups. However, a medium effect was found in the subscale of changes in the relationships with others in benefit finding (*d*_T3-T1_ = 0.42). Medium to large effects were also seen in the total score of personal recovery (*d*_T2-T1_ = 0.53) as well as two subscales in personal recovery, i.e., no domination by symptoms (*d*_T2-T1_ = 0.74, *d*_T3-T1_ = 0.79) and willingness to ask for help (*d*_T2-T1_ = 0.80, *d*_T3-T1_ = 0.42). Additionally, medium effects were observed in subjective well-being (*d*_T3-T1_ = 0.62) and psychiatric symptoms and functional impairment (*d*_T3-T1_ = 0.44) (Table 2).

**Figure 2:**
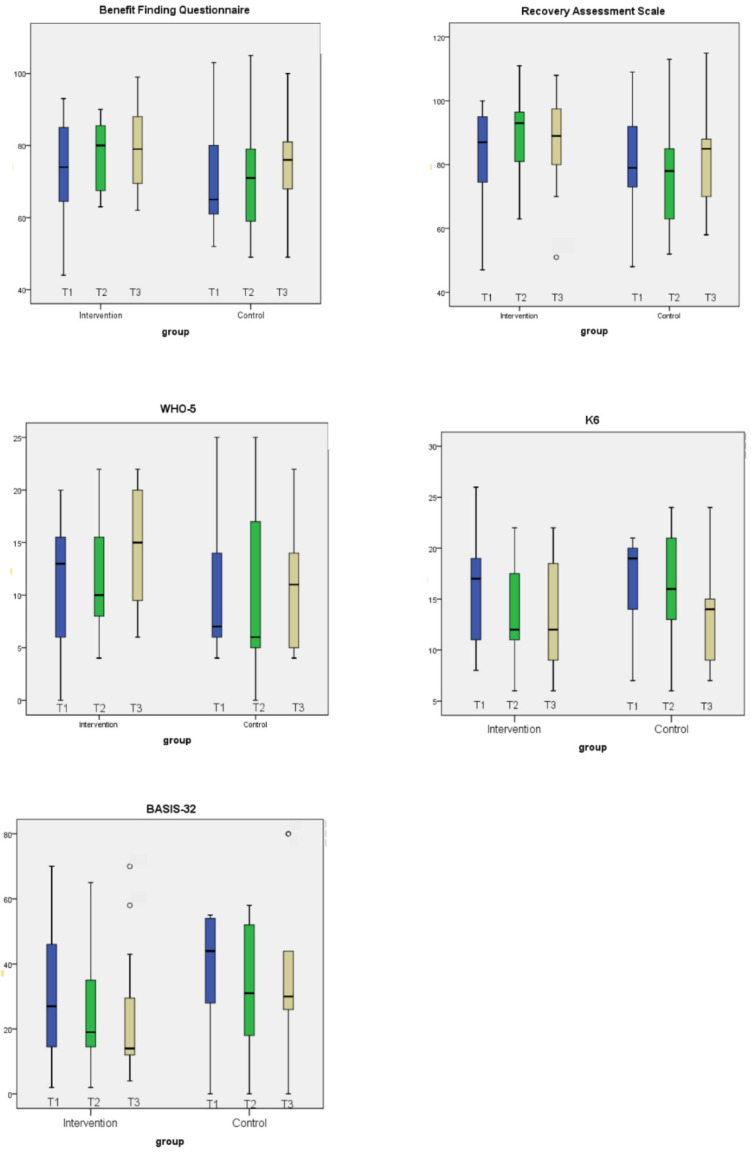
The box plots of the scores in each scale from baseline to T3 (3-month follow up) in intervention group (*n* = 15) and control group (*n* = 9).

Figure 2 shows the changes of the distribution of each score in three time points in each group. In the intervention group, especially those with lower scores improved their scores in benefit finding, personal recovery and subjective well-being; and vice versa in psychological distress, as well as psychiatric symptoms and functional impairment. In the control group, wider ranges of the scores in each time point were observed in benefit finding, personal recovery and subjective well-being, while median values partially increased with time.

## 4. Discussion

This study developed the new program to facilitate benefit finding for people with mental illness and examined its preliminary efficacy using a pilot randomized controlled trial. We were unable to find significant differences over time by groups; however, medium to large effects were observed in the total score or at least a subscale in each scale. The study feasibility seemed adequately high, considering that most of the participants completed the study, except for two who stopped coming to the facility in the middle of the study.

Benefit finding showed its medium effect in follow-up in the relationships with others. The own experiences section is considered helpful in finding new meaning in their experiences, which is consistent with earlier studies [19,20]. Not only thinking about their experiences based on the workbook but also sharing their feelings and experiences with the peer supporter and other participants might be helpful to be aware of their own experiences of benefit finding. The cognitive–behavioral stress management section revealed that participants in the intervention group were encouraged to think about their current social support and eventually noticed various supports from others. In addition, they could enhance a better coping strategy through this group-based intervention. The opportunities to think about how to extend good social support, as well as to improve coping strategy seemed to be effective to strengthen their benefit finding [7,10,33,34]. The process of benefit finding may be naturally experienced by themselves without any specific intervention as observed in previous studies [7], because the control group showed slight and gradual progress in benefit finding. However, as shown in Figure 2, especially for people with fewer experiences of benefit finding, the current intervention is considered to be effective. This study indicates that the benefit-finding process can be facilitated by adequate approaches, including coping strategies. Participants could make use of such strategies in the program even after the intervention, considering the increased effect in the follow-up. Therefore, homework regarding stress management that can be included in daily routine may be effective.

Personal recovery in the intervention group was considerably facilitated after the intervention. Benefit finding and personal recovery are closely related to each other, and personal recovery is partially regarded as a process that results from benefit finding [12,34]. Therefore, finding similar positive changes in both are reasonable. The own experiences section could particularly enhance finding new meanings in their lives, thereby leading to higher personal recovery progress. Meanwhile, domains, such as goal/success orientation and hope, as well as personal confidence, showed slight effects. The study result implies something in common and different effective approaches for facilitating personal recovery and benefit finding, since personal recovery is more future-oriented, while benefit finding is a process to make sense of the past.

Subjective well-being, including good mental health, also considerably improved in the intervention group as earlier studies suggested [35,36]. Subjective well-being may gradually grow through the experience of benefit finding since benefit finding is closely related to better adaptation and sense making. Additionally, participants in the intervention group had the sessions on stress management, including coping, and learned, practiced, and shared their stress management. As earlier studies suggested [15,37], improved stress management could contribute to better subjective well-being. In addition, according to the social cognitive processing theory, the disclosure of one’s own experiences can enable a coherent restructuring of tough memories into existing schemas, which leads to stress relief and improved health [19].

Psychiatric symptoms and functional impairment in the intervention group were proportionally relieved, especially in the follow-up. The stress management section, as well as daily self-check of mental health conditions as homework, seem to be especially helpful in alleviating psychiatric symptoms and improving their mental health. Additionally, these components were easy to continue in daily life even after the intervention period. In addition, the own experiences section might help participants regulate negative emotions, rebuild cognitive structures, and ultimately reduce psychological distress based on the social cognitive processing theory [19].

Overall, each variable changed after intervention as expected. However, the mechanism and reciprocal influences among these concepts in this study remained unclear. Future studies may reveal such details and make the intervention program more effective.

This program was designed and implemented as a group-based intervention with a peer facilitator, except for the homework. As earlier studies for people with mental illness suggested [13,20], having sessions with other participants and a peer with similar illness or experiences seems effective in enhancing benefit finding and personal recovery, since it provides mutual support to share their feelings and experiences and find meaning in their experiences. On the other hand, some people with mental illness may prefer individual intervention due to such as psychiatric symptoms and interpersonal tension. Therefore, future studies using individual sessions may also be needed.

In this study, each session was participated by one peer supporter with many experiences of peer support. We consider that the fidelity of the program was secured by reviewing each session between two facilitators (a psychiatric nurse and a peer supporter) based on the facilitation guide. However, given that experiences with mental illness, including benefit finding, vary from person to person, how peer facilitators concretely talk and what they talk about in the own experience section is not precisely defined in the facilitation guide. Hence, the effectiveness of the intervention may be influenced depending on the peer facilitator’s experience and the expression during the session.

We should mention some limitations in this study. First, the sample size was small due to recruitment issues. As statistical power was not enough to verify the significant differences, we evaluated the effect by effect sizes. Therefore, we cannot conclude the precise effectiveness of this program. In addition to the small sample size, a few other limitations should be addressed. The current study only conducted one follow-up survey 3 months after the intervention. Therefore, the length of the intervention effect is unclear. Additionally, the participants in this study were from only four facilities in one region in Japan. Therefore, we should interpret the result with limited generalizability.

Future RCTs with a larger sample size are surely needed to rigorously examine the program’s effectiveness, including two-factor (intervention and time) interaction. In addition, future studies with longer follow-up periods and varied participants, including inpatients, should also be considered. Future studies may reveal the mechanism of how these components of the program can have a synergistic effect on benefit finding. Despite these limitations, the current study sounds meaningful enough, as it indicated two approaches, i.e., (1) cognitive–behavioral stress management and (2) own experiences, including what was found or realized through their lives since the onset of mental illness, that can enhance benefit finding.

## 5. Conclusions

This study developed a group-based intervention program to facilitate benefit finding for people with mental illness and examined its preliminary efficacy. The program included cognitive–behavioral stress management and own experiences sections. Medium to large effects in each scale or at least one subscale (i.e., benefit finding, personal recovery, subjective well-being, and psychiatric symptoms and functional impairment) were observed. However, future studies with more participants from various settings would be necessary to exactingly examine the effectiveness of the intervention program.

## Figures and Tables

**Table 1:** Clinical and sociodemographic characteristics of the participants in the study (*n* = 24).

	Total (*n* = 24)		Intervention (*n* = 15)		Control (*n* = 9)	
**Characteristic**	*n*/Mean	%/*SD*	*n*/Mean	%/*SD*	*n*/Mean	%/*SD*
**Gender** (male), *n* (%)	11	(45.8)	9	(60.0)	2	(22.2)
**Age** (years), Mean (*SD*)	42.5	(13.4)	39.0	(11.6)	48.3	(14.7)
Range	22–72		22–56		22–72	
** Duration of mental illness** (years), Mean (*SD*)	16.3	(9.9)	15.0	(6.5)	18.4	(14.1)
Range	3–44		5–26		3–44	
**Diagnosis**, *n* (%)						
Schizophrenia	15	(62.5)	10	(66.7)	5	(55.6)
Depression	6	(25.0)	3	(20.0)	3	(33.3)
Bipolar disorder	2	(8.3)	1	(6.7)	1	(11.1)
Other	1	(4.2)	1	(6.7)	0	(0.0)
** Experience of hospitalization for psychiatric wards** (Yes), *n* (%)	6	(25.0)	4	(26.7)	7	(77.8)
** Duration of hospitalization for psychiatric wards** (months), Mean (*SD*)	32.7	(96.5)	10.3	(10.3)	70.1	(155.1)
Range	0–481		0–30		0–481	
**Recipient of Mental disability certificate** (Yes), *n* (%)	23	(95.8)	14	(93.3)	9	(100.0)
**Recipient of Disability pension** (Yes), *n* (%)	12	(50.0)	8	(53.3)	4	(44.4)
**Recipient of Livelihood protection** (Yes), *n* (%)	10	(41.7)	7	(46.7)	3	(33.3)
**Services in use** (multiple answers), *n* (%)						
Employment support (Yes)	18	(75.0)	14	(93.4)	4	(44.4)
Community activity support center (Yes)	4	(16.7)	3	(20.0)	1	(11.1)
Home-visit nursing (Yes)	6	(25.0)	4	(26.7)	2	(22.2)
Psychiatric day care center (Yes)	4	(16.7)	2	(13.3)	2	(22.2)
Self-help group (Yes)	3	(12.5)	1	(6.7)	2	(22.2)
**Experience as a peer** (Yes), *n* (%)	8	(33.3)	4	(26.7)	4	(44.4)

**Table 2:** The changes of the scores in each scale from baseline to 3-month follow up, with differences within groups over time (*n* = 24).

	Intervention (*n* = 15)						Control (*n* = 9)						Test of Difference					
	Mean	(*SD*)	Mean Change *(SD)* from T1				Mean	*(SD)*	Mean Change *(SD)* from T1				T2-T1			T3-T1		
**Instruments and subscales** (range)	T1		T2		T3		T1		T2		T3		*t*	*df*	Effect Size (*d*)	*t*	*df*	Effect Size (*d*)
**Benefit Finding Questionnaire (21–105)**	72.9	(14.2)	4.9	(16.4)	6.6	(15.8)	71.2	(15.9)	1.1	(5.9)	3.9	(9.1)	0.8	19	0.28	0.5	22	0.20
Changes in sense of values and way of thinking (13–65)	45.6	(10.8)	3.1	(11.7)	3.8	(11.0)	44.1	(11.6)	0.3	(3.4)	3.3	(7.7)	0.9	18	0.29	0.1	22	0.05
Changes in relationships with others (8–40)	27.3	(4.5)	1.8	(5.6)	2.8	(6.0)	27.1	(5.0)	0.8	(3.0)	0.6	(3.8)	0.5	22	0.21	1.0	22	**0.42**
**Recovery Assessment Scale** **(24–120)**	83.0	(15.6)	5.9	(13.2)	3.8	(11.5)	80.0	(21.6)	−0.3	(8.7)	2.2	(14.2)	1.3	22	**0.53**	0.3	22	0.13
Goal/success orientation and hope (9–45)	32.3	(7.9)	1.3	(6.0)	1.9	(5.1)	30.8	(7.5)	−0.1	(4.8)	1.7	(6.5)	0.6	22	0.26	0.1	22	0.05
Reliance on others (4–20)	14.5	(3.5)	0.7	(1.8)	−0.7	(2.1)	14.4	(3.9)	0.0	(1.6)	−0.6	(2.6)	0.9	22	0.38	−0.1	22	0.05
Personal confidence (5–25)	15.1	(4.0)	2.1	(4.7)	1.5	(3.2)	13.4	(5.6)	1.0	(3.0)	2.4	(4.2)	0.7	22	0.22	−0.6	22	0.27
No domination by symptoms (2–10)	6.9	(1.8)	0.5	(1.5)	0.6	(1.5)	7.4	(1.9)	−0.6	(1.2)	−0.8	(2.1)	1.8	22	**0.74**	1.8	22	**0.79**
Willingness to ask for help (4–20)	14.2	(2.3)	1.3	(2.5)	0.5	(2.5)	13.9	(3.9)	−0.7	(2.2)	−0.6	(2.2)	1.9	22	**0.80**	1.0	22	**0.42**
**WHO-5 Well-Being Index (0–25)**	11.1	(6.1)	1.0	(5.2)	3.4	(6.1)	11.0	(7.7)	−0.7	(9.2)	0.1	(3.6)	0.6	22	0.24	1.5	22	**0.62**
**BASIS-32 (0–128)**	31.2	(20.5)	-5.1	(21.1)	-8.1	(20.5)	37.0	(21.4)	−4.1	(10.0)	0.7	(19.3)	−0.1	22	0.05	-1.0	22	**0.44**

Note. T1; Baseline. T2; Post-intervention (eight weeks after baseline). T3; Three-month follow-up after the post-intervention. All Ps were larger than 0.05.
